# Dissolving microneedles incorporating kopexil multicomponent crystals for improved transdermal delivery

**DOI:** 10.1007/s13346-025-02042-0

**Published:** 2026-01-28

**Authors:** Yuehua Deng, Si Nga Wong, Wing Chi Nico Chan, Xinyue Zhang, Shing Fung Chow

**Affiliations:** 1 Department of Pharmacology and Pharmacy, Li Ka Shing Faculty of Medicine, The University of Hong Kong, L2-08B, 2/F, Laboratory Block, 21 Sassoon Road, Pokfulam, Hong Kong SAR China; 2 Advanced Biomedical Instrumentation Centre, Hong Kong Science Park, Shatin, New Territories, Hong Kong SAR China

**Keywords:** Kopexil, Multicomponent crystals, Microneedle, Transdermal delivery, Androgenetic alopecia

## Abstract

Notwithstanding the rapid advancements in microneedle research, the applications of multicomponent crystals (MCCs) within microneedle drug delivery landscape remain largely underexplored. This study aimed at integrating crystal engineering with powder-carrying microneedle technology to overcome the current therapeutic obstacles in androgenetic alopecia (AGA). Kopexil (KPX), a minoxidil analog for AGA treatment, was successfully synthesized into MCCs with benzoic acid (BA) and salicylic acid (SA), yielding KPX-BA·H_2_O cocrystal hydrate and KPX-SA·H_2_O salt hydrate. The solid-state properties of the resulting MCCs were thoroughly characterized. Single-crystal analysis indicated that MCCs were stabilized by acid-aminopyrimidine heterosynthons. The KPX MCC powders were further formulated into dissolving microneedles (MNs). In vitro membrane diffusion tests demonstrated KPX MCCs encapsulated in MNs achieved a 1.86-fold and a 3.20-fold reduction in the amounts of KPX diffused compared to KPX·H_2_O at 4 h. The diffusion of KPX MCCs was positively correlated with their solubilities, following the first-order kinetic model. With the absence of prior reports on KPX MCCs, these findings can inform the broader development of MCCs for APIs that have yet to be explored. The use of pharmaceutically accepted coformers, scalable fabrication methods, predictable release profiles, favorable biocompatibility, and painless self-application microneedle patches collectively offer a viable strategy for the potential clinical translation of this platform for AGA.

**Supplementary Information:**

The online version contains supplementary material available at https://doi.org/10.1007/s13346-025-02042-0.

## Highlights

- Rational design and synthesis of the first kopexil multicomponent crystals.
- Novel delivery platform integrating crystal engineering and microneedle technology.
- Dissolving microneedles for preserving the solid-state integrity of KPX MCCs.
- Attained sustained drug release to improve therapeutic outcomes for hair regrowth.

**Graphical abstract:**

**Supplementary Information:**

The online version contains supplementary material available at https://doi.org/10.1007/s13346-025-02042-0.

## Graphical abstract

**Figure:**
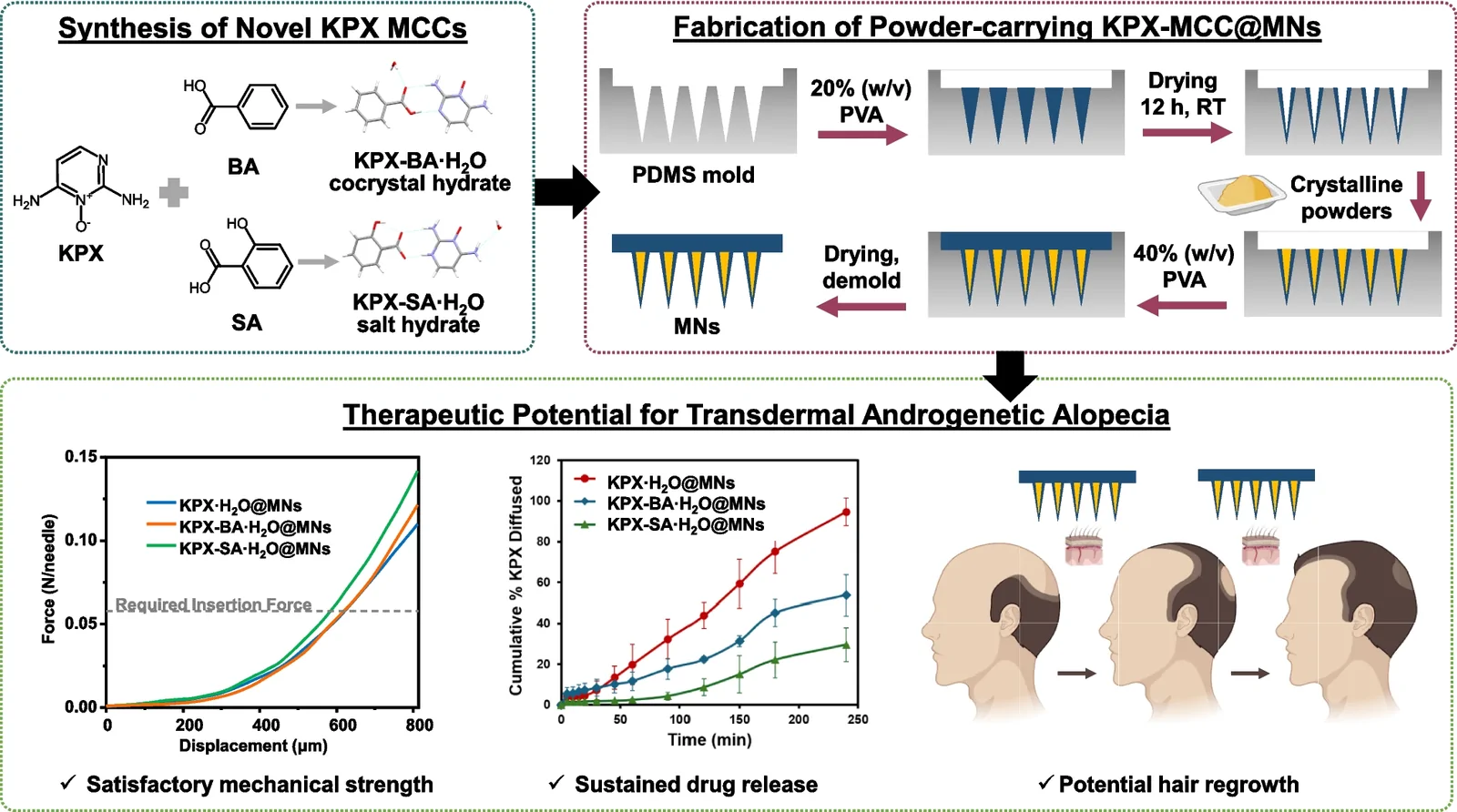

## Introduction

Androgenetic alopecia (AGA), the most prevalent form of hair loss, afflicts at least 80% of males and 50% of females by age 70 years [1]. Contemporary societal trends indicate an escalating incidence of AGA in younger adults [2], highlighting the critical need for effective therapeutic interventions. The management of AGA remains a medical challenge, with currently approved treatment options in the U.S. limited to topical minoxidil, oral finasteride, and low-level light therapy [3]. Recent studies have highlighted the role of kopexil (KPX), a structural analog of minoxidil, as a novel alternative designed to address the safety concerns associated with conventional AGA treatments [4, 5]. Despite the reduced toxicity, the hydrophilic nature of KPX restricts its cutaneous permeation across the lipophilic barrier of the stratum corneum, thereby hindering the full realization of its therapeutic potential [6]. In this study, an integrated combining crystal engineering and microstructural design will be employed to formulate KPX as multicomponent crystals (MCCs) into microneedle patches. This approach facilitates targeted drug delivery to skin capillaries for hair regrowth while simultaneously modifying the pharmaceutical properties of KPX.

Microneedle-based products breach the skin barrier for transdermal delivery of active pharmaceutical ingredients (APIs) [7]. While dissolvable MNs are favored over solid MNs due to reduced injury risks and enhanced dosing [8, 9], conventional fabrication involving aqueous solvents is ill-suited for solid-state MCC powders. This limitation arises from the inherent risk of solution-mediated phase transformation (e.g., cocrystal dissociation and salt disproportionation) occurred during the micro-casting process [10, 11] and thus impeding their successful advancement into practical application in the domain of microneedle technology. To preserve MCC integrity, a powder-carrying dissolving MNs strategy that directly encapsulates KPX MCCs to MN cavities in a dry powder form is deemed the most ideal for the purpose of the present study [12, 13]. It combines the advantages offered by both dissolvable MNs and solid-state APIs, effectively attenuates the unfavorable MCC-solvent or MCC-polymer interactions, improving stability during fabrication and shelf-life.

The goal of this study is to develop a powder-carrying MN formulation incorporating KPX MCC for transdermal hair regrowth therapy. To our knowledge, no pharmaceutical cocrystals or salts of KPX have been reported. This study aims: i) to synthesize and characterize novel KPX MCCs using aromatic carboxylic acids; ii) to fabricate polyvinyl alcohol (PVA)-based MNs incorporating KPX MCC powders; and iii) to determine their mechanical strength, physical stability, drug release profile, and in-vitro cytotoxicity. Two generally recognized as safe (GRAS) compounds, salicylic acid (SA) and benzoic acid (BA) were selected as coformers. Beyond their antimicrobial and keratolytic properties [14–16], these agents offer synergistic benefits with KPX [17, 18], including reduced follicular keratinization, bacterial inhibition, and enhanced permeation.

## Materials and methods

### Materials

KPX (98%, CAS#:74638–76-9) and rhodamine B (AR) were supplied by Shanghai Macklin Biochemical co. ltd (China). BA (purity ≥ 99.5%, CAS#: 65–85-0), SA (purity ≥ 99.5%, CAS#: 69–72-7) and PVA (98–99% hydrolyzed, MW 31–50 kDa) were obtained by Sigma-Aldrich (USA). Methanol (MeOH), ethanol (EtOH) and acetonitrile (ACN) of analytical grade were purchased from Merck KGaA (Germany). Acetic acid glacial (purity ≥ 99.7%, HPLC grade) was supplied by Fisher Chemical (USA). Potassium bromide was sourced from J&K Scientific Limited (China). Ultra-purified water was generated using a Barnstead NANOpure Diamond system (Thermo Fisher Scientific, Waltham, MA, USA).

### Computational analysis

#### Bravais–Friedel–Donnay–Harker (BFDH), Full Interactions Map (FIM), and water interaction map

The morphologies of single crystals were predicted from the structural data using the geometric method BFDH [19] model in both Mercury 2024.3 [20] and Biovia Materials Studio 2023 (Accelrys, San Diego, USA). The FIM [21] and water interaction map calculations were performed in Mercury 2024.3.

#### Molecular Electrostatic Potential (MEP) surfaces

All molecular structures were geometrically optimized with the B3LYP/6-31g** basis set in Gaussian 16 software [22]. The Multiwfn 3.8 program [23, 24] was employed to analyze the MEP mapped onto the 0.001 Bohr Å^–3^ electron density isosurface for illustrating the distribution of the global extrema. Visual Molecular Dynamics (VMD) v.1.9.3 software [25] was used to visualize the simulation results.

#### Hirshfeld Surface (HS) analysis

The detailed information on the molecular interactions in crystal structures was visualized and quantified through Hirshfeld surface analysis and 2D fingerprint plots using CrystalExplorer 17.5 software [26], revealing the intermolecular interactions during crystal assembly and quantifying the contribution of these interactions in the distinct crystal packing.

### Preparation of the KPX MCCs

The purchased KPX was in monohydrate form and was dehydrated in an oven at 170 °C prior to the preparation of KPX MCC. To synthesize KPX MCCs, equimolar quantities of KPX (37.8 mg, 0.3 mol) and the selected coformers (SA and BA) were initially dissolved in either MeOH or EtOH with vigorous magnetic stirring for 20 min. The resulting solution was then filtered through a 0.22 μm nylon membrane to remove impurities. Subsequently, the solvent was removed through evaporation in a fume hood, and the residual solid was subjected to solid-state characterizations. To obtain single crystals suitable for single crystal X-ray diffraction analysis, the solution was allowed to evaporate gradually at room temperature to facilitate the formation of well-ordered crystalline structures.

### Solid-state characterization methods

**Powder X-Ray Diffraction (PXRD)** was performed using a Rigaku SmartLab 9 kW diffractometer (Tokyo, Japan) equipped with a Cu-Kα radiation source (*λ* = 1.54056 Å) operating at 45 kV and 200 mA. Samples were uniformly packed in a quartz sample holder and scanned over a 2*θ* range of 5°–35° with a step size of 0.02° and a scanning rate of 5° min⁻^1^.

**Differential Scanning Calorimetry (DSC)** was conducted to assess thermal behavior and phase purity using a TA DSC 250 (New Castle, DE, USA). The instrument was calibrated using an indium standard. Samples (3–5 mg) were accurately weighed and encased into aluminum Tzero pans covered with pinhole lids. These samples were heated from 35 °C to 300 °C at a ramp rate of 5 °C min⁻^1^, under a nitrogen purge of 50 mL min⁻^1^ to maintain an inert environment.

**Thermogravimetric analysis (TGA)** was carried out to examine sample weight loss during heating. Samples were placed in platinum pans and heated from room temperature to 300 °C at a heating rate of 10 °C min⁻^1^ using a TA TGA250 (New Castle, DE, USA). Thermal transitions were recorded and analyzed with TA TRIOS software (v5.1.1).

**Fourier-transform infrared (FTIR)** spectroscopy was performed using a Bruker ALPHA FTIR spectrophotometer (Ettlingen, Germany) equipped with a diffuse reflectance accessory. Samples were mixed with anhydrous potassium bromide (KBr) at an approximate 1:100 (w/w) ratio and compressed into 13-mm pellets using a Graseby Specac manual hydraulic press (Orpington, UK) under a uniaxial pressure of 2 tons. Spectra were acquired in the mid-infrared region (600–3,500 cm⁻^1^) at a resolution of 4 cm⁻^1^, with 64 scans per measurement. Background correction was done using a pure KBr pellet prior to sample analysis.

**Single crystal X-ray diffraction (SCXRD)** data were collected using a Bruker D8 Quest diffractometer (Karlsruhe, Germany) equipped with a Mo Kα radiation source (*λ* = 0.71073 Å). Initial crystal indexing and data integration were carried out using the Difference Vectors method in APEX3 (Bruker AXS, 2019). A multiscan absorption correction was applied using SADABS. Space group determination was accomplished via XPREP (integrated in APEX3). The structures were solved by direct methods using SHELXL-2014 and refined by full-matrix least-squares minimization on F^2^ with anisotropic displacement parameters for all non-hydrogen atoms. Hydrogen atoms bound to carbon and nitrogen were placed in idealized positions and refined isotropically with Uiso(H) = 1.2 × *U*eq (C/N).

**Crystal morphology** was examined using a Hitachi S-3400N scanning electron microscope (SEM). The surface morphology of the MNs was visualized using both SEM and a Nikon SMZ745T stereoscopic microscope. To minimize charging artifacts during SEM imaging, the samples were coated with a thin layer of gold.

### High performance liquid chromatography (HPLC)

An Agilent 1200 HPLC system (Wilmington, DE, USA), equipped with a diode array detector and an Agilent Zorbax Eclipse Plus C18 column (5 μm, 250 mm × 4.6 mm), was employed for KPX assay. Samples were eluted isocratically using a mobile phase composed of ACN, water, and acetic acid [58:40:2 (v/v/v)], at a flow rate of 1 mL/min. Five microliters of samples were injected into the column. The wavelength for KPX detection was set at 273 nm, with a retention time of 2.2 min. The calibration curve displayed linearity with an *R*^*2*^ > 0.9999 over the concentration range of 0.01–1 mg/mL.

### Equilibrium solubility determination

Drug solubility of KPX was measured by adding excess solid in screw-capped test tubes with 3 mL of pH 5.5 phosphate buffer solution under ambient conditions. The mixtures were agitated for 72 h to attain equilibrium. Subsequently, the samples were filtered through a 0.45 μm pore membrane and diluted to appropriate concentrations for HPLC analysis. The residual solid phases were characterized by PXRD to verify their phase identity.

### Fabrication of the KPX MCC-loaded dissolving microneedles (MNs)

MNs were fabricated via a mold-casting technique according to a reported protocol [13]. The microneedle arrays were produced using polydimethylsiloxane (PDMS) molds containing 100 conically tapered microstructures arranged in a 10 × 10 matrix. Each microneedle has a height of 900 μm, a base diameter of 400 μm, a tip diameter of 5 μm, and an inter-needle spacing of 1,000 μm. The microneedle shells were formed using an aqueous solution of 20% (w/v) PVA. To construct the hollow cavities, 0.5 mL of the PVA solution was poured into the mold grooves and subjected to centrifugation (3,000 rpm (1,690 × g), 6 min, 25 °C). The residual polymer from the mold surface was carefully removed using a small, flat laboratory spatula, and the molds were subsequently dried in a fume hood for 12 h at room temperature. As water evaporated, the PVA solution solidified, yielding hollow conical structures. The powders of KPX·H_2_O, KPX-BA·H_2_O and KPX-SA·H_2_O were ground using a mortar and pestle to ensure particle homogeneity. They were then loaded into microneedle cavities through centrifugal compaction (5,000 rpm (4,696 × g), 10 min). A backing layer was created by depositing 0.3 mL of a 40% (w/v) PVA solution over the groove surface, followed by desiccation (12 h, 25 °C). The fully dried MNs were carefully demolded and stored in hermetically sealed 6-well plates to prevent moisture absorption.

### Mechanical strength and in vitro skin insertion study

The mechanical properties of the MN arrays were evaluated using a TA-XT2 Texture Analyser (Stable Microsystems, Haslemere, UK) operating in compression mode. A test distance of 810 μm (corresponding to 90% compression) was applied, with a trigger force of 0.049 N. The pre- and post-test speeds were maintained at 1.0 mm/s, while the testing speed was set to 0.5 mm/s. During testing, the MN tips were oriented upward and subjected to axial compression using the texture analyzer. The resulting force measurements were used to generate a force–displacement curve.

The mechanical integrity and insertion performance of the KPX MCC loaded MNs were assessed using an in vitro porcine skin model [27, 28]. Skin samples were prepared by removing subcutaneous tissue and hair, trimmed to a thickness of 1 mm, and sectioned into 20 × 20 mm pieces. After cleaning with PBS and drying, MN arrays with a rhodamine B-loaded PVA shell were applied to the fixed skin using a 20 N force for 60 s. Insertion sites were subsequently examined with an optical microscope, and the percentage of insertion efficiency was calculated using Eq. (1):

1 $$\%\, insertion\, efficiency=\frac{Number \,of\, stained \,red \,dots}{Number\, of \,needles \,on\, each \,patch \,(100)}\times 100$$

### Drug content analysis of MNs

The drug content in the MN arrays loaded with KPX·H_2_O, KPX-BA·H_2_O, and KPX-SA·H_2_O was assessed in triplicate by dissolving individual MN arrays into a glass vial containing 2 mL of water, followed by sonication for 15 min. The solution was then diluted with 2 mL of MeOH and further subjected to sonication for 15 min. 100 μL of the sample was transferred into a 1.5-mL microcentrifuge tube and mixed with 0.9 mL ACN to precipitate the polymeric matrix while the drug remained dissolved [29]. The resulting dispersion was then centrifuged at 6,500 rpm (3,117 × g) for 30 min. The supernatant was collected for HPLC analysis.

### In vitro drug release (membrane permeation) and drug deposition study

The in vitro drug release profiles from the MN arrays loaded with KPX·H_2_O, KPX-BA·H_2_O, and KPX-SA·H_2_O was assessed in triplicate using a Franz diffusion cell with a 9 mm orifice diameter and a 5 mL receptor volume (PermeGear, Hellertown, PA, USA) according to the reported protocol [12, 30]. The MN arrays, with numbers of needles containing equivalent amount of KPX (approximately ∼ 0.47 mg) in all groups, were inserted into single layer of Parafilm M^®^ (Amcor, USA) as an artificial skin model [31], and adhesive tape was carefully applied on the upper surface to secure the position. The Parafilm M^®^ with the MN arrays inserted was mounted between the donor and receptor compartments of the Franz diffusion cell with an effective permeation area of 1 cm^2^ and affixed with a pinch clip. A metallic weight of 6 g was placed onto the upper surface of the MN array to prevent expulsion during the experiment. The receptor compartment was filled with pH 5.5 buffer solution, and the temperature was controlled at 32 ± 0.1 °C mimicking the skin surface temperature by a water circulator [32]. The receptor medium was stirred constantly at 500 rpm with a magnetic bar. The donor compartment and sampling port were covered with Parafilm M^®^ to minimize evaporation. Sink conditions were maintained throughout the experiment. At specific time points of 5, 10, 15, 20, 30, 45, 60, 90, 120, 150, 180, 240 min, 400 μL of dissolution medium was withdrawn and replenished with an equal volume of fresh medium. The samples were assayed for KPX content by HPLC. The cumulative amount (Q, μg/cm^2^) of KPX permeated through membrane was calculated by the Eq. (2) [33]:

2 $$Q=\frac{{C}_{n}\times V+0.4\times {\sum }_{i=10}^{n-1}{C}_{i}}{S}$$

where S is the effective area (cm^2^), V is the volume of receptor cell (mL), ${C}_{n}$ is the KPX concentration at time point ‘n’ (μg/mL), and ${C}_{i}$ is the KPX concentration at time point ‘i’ (μg/mL). The collected data were fitted to zero-order kinetics, first-order kinetics, Higuchi model and Korsmeyer–Peppas model to characterize the drug release mechanism [34]. The goodness of fit for these models was determined using the squared correlation coefficient (R^2^).

Determination of KPX deposited in porcine skin was performed using the same Franz diffusion cell setup as in vitro drug release study with porcine skin replacing Parafilm M^®^ [27, 28]. After 12 h, the skin was detached and cut into small pieces. The drug deposited in the skin was extracted using MeOH. The sample solution was centrifuged at 13,000 × g for 10 min to precipitate the skin and residue. Finally, the supernatant was quantified using HPLC. The percentage of drug deposition was calculated following Eq. (3):

3 $$\%\, Drug \,deposition=\frac{Amount \,of \,drug\, in \,the\, porcine\, skin}{Amount \,of \,drug\, in \,the\, formulation}\times 100$$

### Stability study

To study the stability of the powders within MN patches, KPX·H_2_O@MNs, KPX-BA·H_2_O@MNs, and KPX-SA·H_2_O@MNs were stored for one month under the conditions of 25 °C/60% RH and 40 °C/75% RH, followed by PXRD characterization.

## Results and discussion

### Solid-state characterizations

**Fig. 1:**
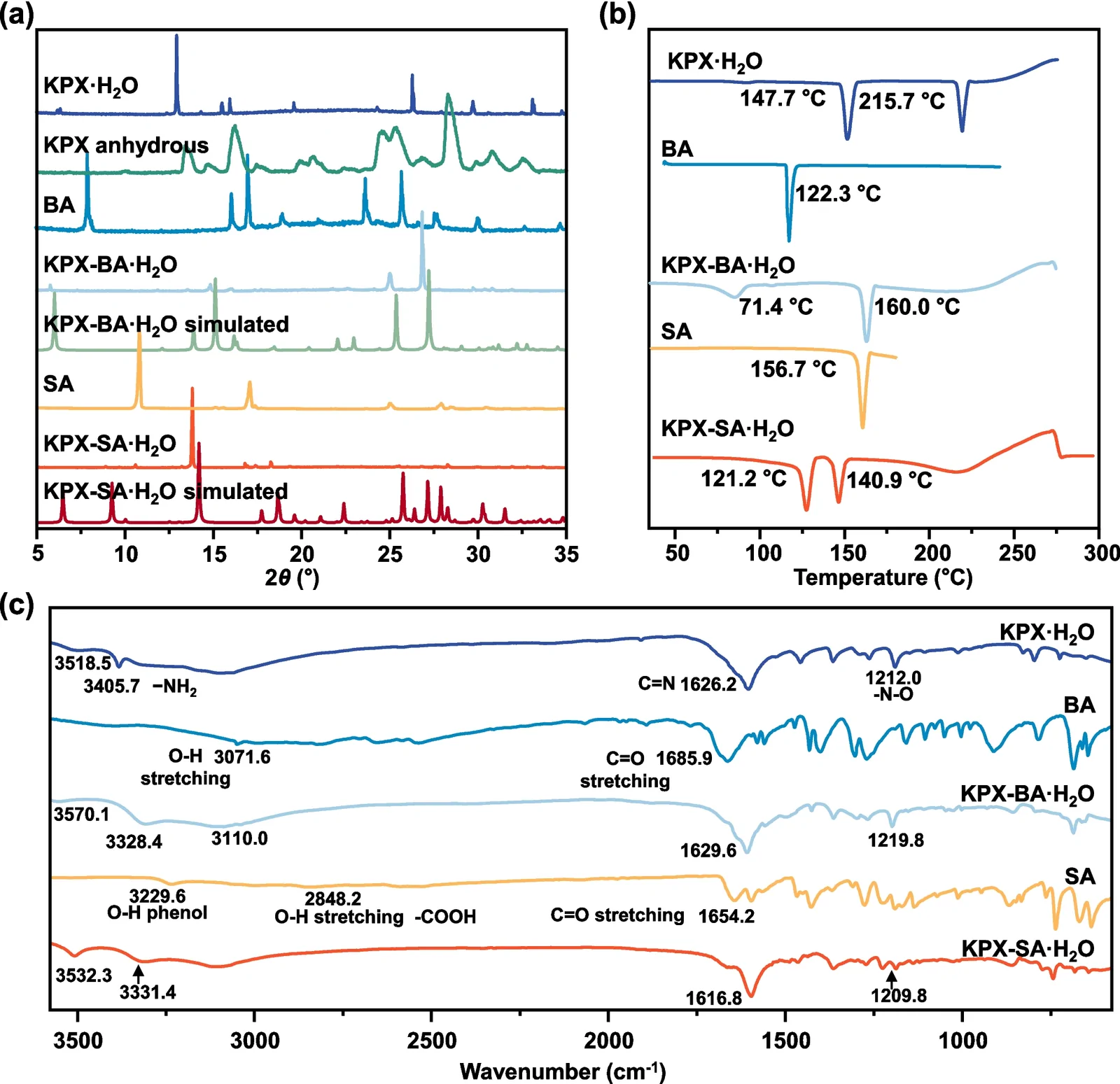
Solid-state characterization of the KPX MCCs and their parent constituents: (**a**) PXRD patterns, (**b**) DSC curves, and (**c**) FTIR spectra

Attempts were made to synthesize KPX MCCs with two aromatic carboxylic acids, namely BA and SA via slow solvent evaporation, resulting in successful formation of new KPX-BA·H_2_O (cocrystal hydrate) and KPX-SA·H_2_O (salt hydrate). From PXRD patterns** (**Fig. 1a), the KPX·H_2_O sample exhibits characteristic diffraction peaks at 6.25°, 6.40°, 13.00°, 14.38°, 15.57°, 19.64°, and 24.37°. The anhydrous KPX sample displays shifted peak positions and reduced crystallinity, suggesting that dehydration substantially alters the crystal structure. Upon the formation of MCCs, KPX-BA·H_2_O produces reflections at 5.84°, 14.91°, 16.13°, 25.10°, and 26.90°, whereas KPX-SA·H_2_O exhibits peaks at 10.67°, 13.89°, 16.88°, 17.47°, 18.34°, and 28.35°. The PXRD patterns of the MCCs are in accordance with the simulated patterns obtained from single-crystal data, confirming successful MCC formation.

The thermal properties of the crystals were analyzed using DSC (Fig. 1b) and TGA (Figure S1). For KPX·H_2_O, two endotherms were observed at 147.7 °C (Δ*H*_f_ = 37.9 kJ/mol) and 215.7 °C (Δ*H*_f_ = 21.1 kJ/mol), corresponding to dehydration and melting of the KPX anhydrous form. Similarly, KPX-BA·H_2_O displayed dehydration at 71.4 °C (Δ*H*_f_ = 25.9 kJ/mol) and melting at 160.0 °C (Δ*H*_f_ = 37.1 kJ/mol), whereas KPX-SA·H_2_O dehydrated at 121.2 °C (Δ*H*_f_ = 60.8 kJ/mol) and melted at 140.9 °C (Δ*H*_f_ = 26.9 kJ/mol). TGA analysis further confirmed the dehydration process, with experimental mass losses of 12.8%, 7.5%, and 7.0% for KPX·H_2_O, KPX-BA·H_2_O, and KPX-SA·H_2_O, respectively, aligning closely with theoretical values of 12.5%, 6.8%, and 6.4%.

The FTIR spectra (Fig. 1c) of the raw drugs and KPX MCCs revealed notable spectral shifts in the vibrational frequencies of key functional groups, indicating altered molecular interactions upon the KPX MCC formation. In the raw KPX·H_2_O, the − NH_2_, C = N, and − N − O stretches appeared at 3,405.7 cm⁻^1^, 1,626.2 cm⁻^1^, and 1,212.0 cm⁻^1^, respectively, while the O − H_water_ vibration was observed at 3,518.5 cm⁻^1^. The carboxylic acid groups of BA and SA exhibited − C = O and O − H stretches at 1,685.9/3,071.6 cm⁻^1^ and 1,654.2/2,848.2 cm⁻^1^, respectively. The stretching frequencies of − NH_2_ manifested as broad peaks at 3,328.4 cm⁻^1^ and 3,331.4 cm^−1^ in KPX-BA·H_2_O and KPX-SA·H_2_O, respectively, which were attributed to the formation of N–H⋯O intermolecular hydrogen bonding between KPX and the respective coformers. In addition, the C = N (KPX) and − C = O (coformers) peaks merged into single wide bands at 1,685.9 cm⁻^1^ (KPX-BA·H_2_O) and 1,616.8 cm⁻^1^ (KPX-SA·H_2_O). The presence of new O − H_water_ vibrations at 3,570.1 cm⁻^1^ (KPX-BA·H_2_O) and 3,532.3 cm⁻^1^ (KPX-SA·H_2_O) confirmed the incorporation of water molecules into the crystal structures. It is worth noting that, upon the MCC formation, the O − H stretching (− COOH) remained detectable in KPX-BA·H_2_O but was absent in KPX-SA·H_2_O. This discrepancy suggests cocrystal formation with BA and salt formation with SA.

### FIM and MEPs analyses

**Fig. 2:**
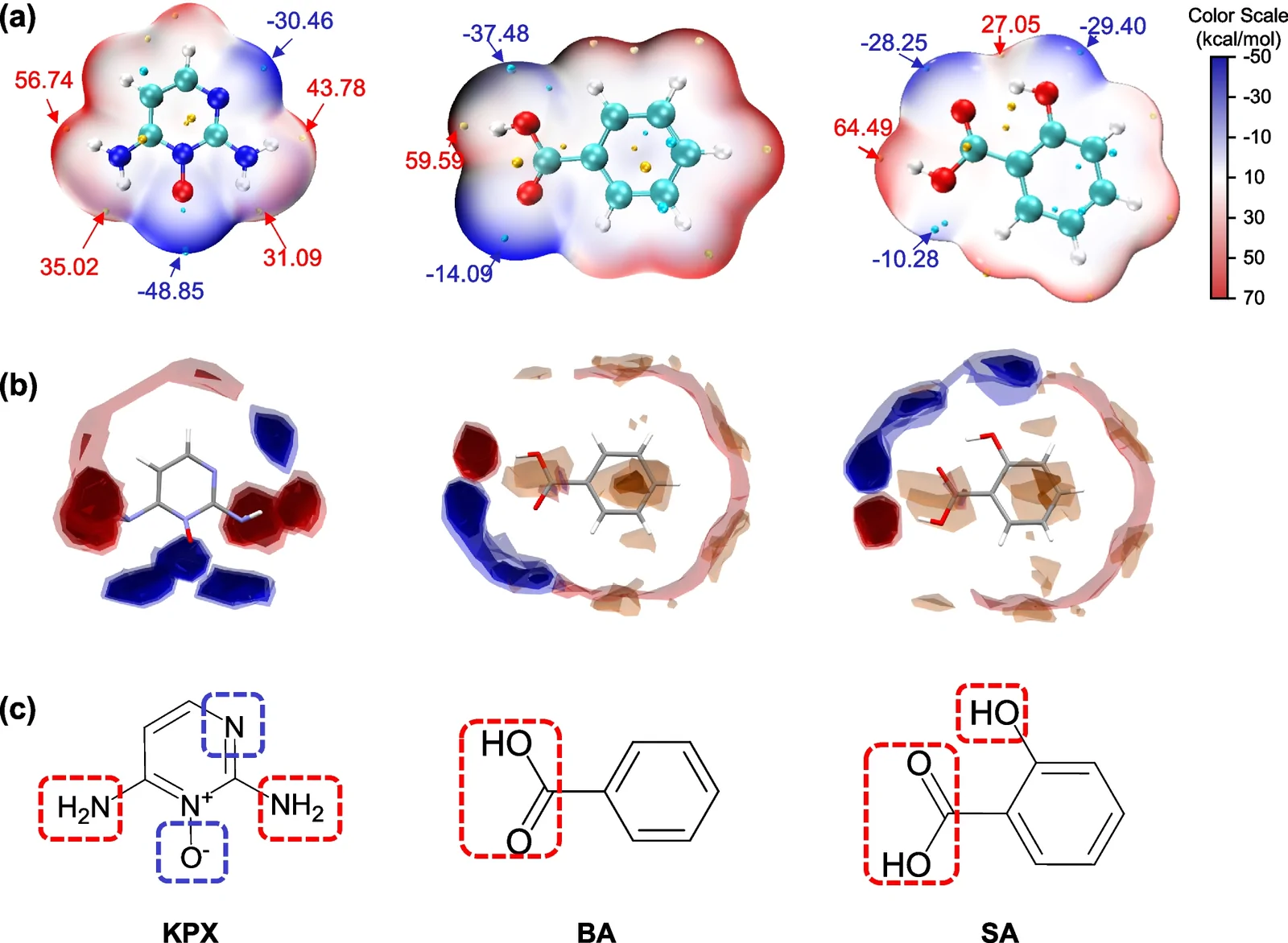
(**a**) MEP surface with extremal points (kcal/mol) visualized as spheres (red = maxima, blue = minima) and numerically labeled in corresponding colors, (**b**) FIM showing potential interaction sites, where blue regions indicate hydrogen bond acceptors, red regions denote hydrogen bond donors, and brown regions represent hydrophobic/aromatic interaction zones, and (**c**) Molecular structures with highlighted interaction sites, where red-outlined functional groups indicate hydrogen bond donors and blue-outlined groups represent acceptors

The spatial distribution of intermolecular interaction sites between KPX and its coformers was visualized through combined FIM and MEP mapping (Fig. 2). The amino groups (56.74 and 43.78 kcal/mol) of KPX, carboxyl group (59.59 kcal/mol) of BA, carboxyl group (64.49 kcal/mol) and hydroxyl group (27.05 kcal/mol) of SA were identified as red regions in both FIM and MEPs maps, implying their potential roles as hydrogen bond donors. Conversely, the pyrimidine (−30.46 kcal/mol) and pyrimidine-N-oxide (−48.85 kcal/mol) moieties of KPX are depicted as blue regions in the maps, signifying their potential as hydrogen bond acceptors. Furthermore, the benzene rings of BA and SA are shown as brown regions in FIM maps, indicating the likelihood of π–π interactions. These computationally predicted interaction patterns are consistent to our experimental findings, thereby validating the application of FIM and MEP in predicting supramolecular synthons and optimizing coformer selection in the design of MCCs. Such computational methodologies can significantly advance the screening and rational design of novel multicomponent crystals for APIs lacking previously reported MCCs, as demonstrated here with KPX.

### Crystal structure analysis

**Fig. 3:**
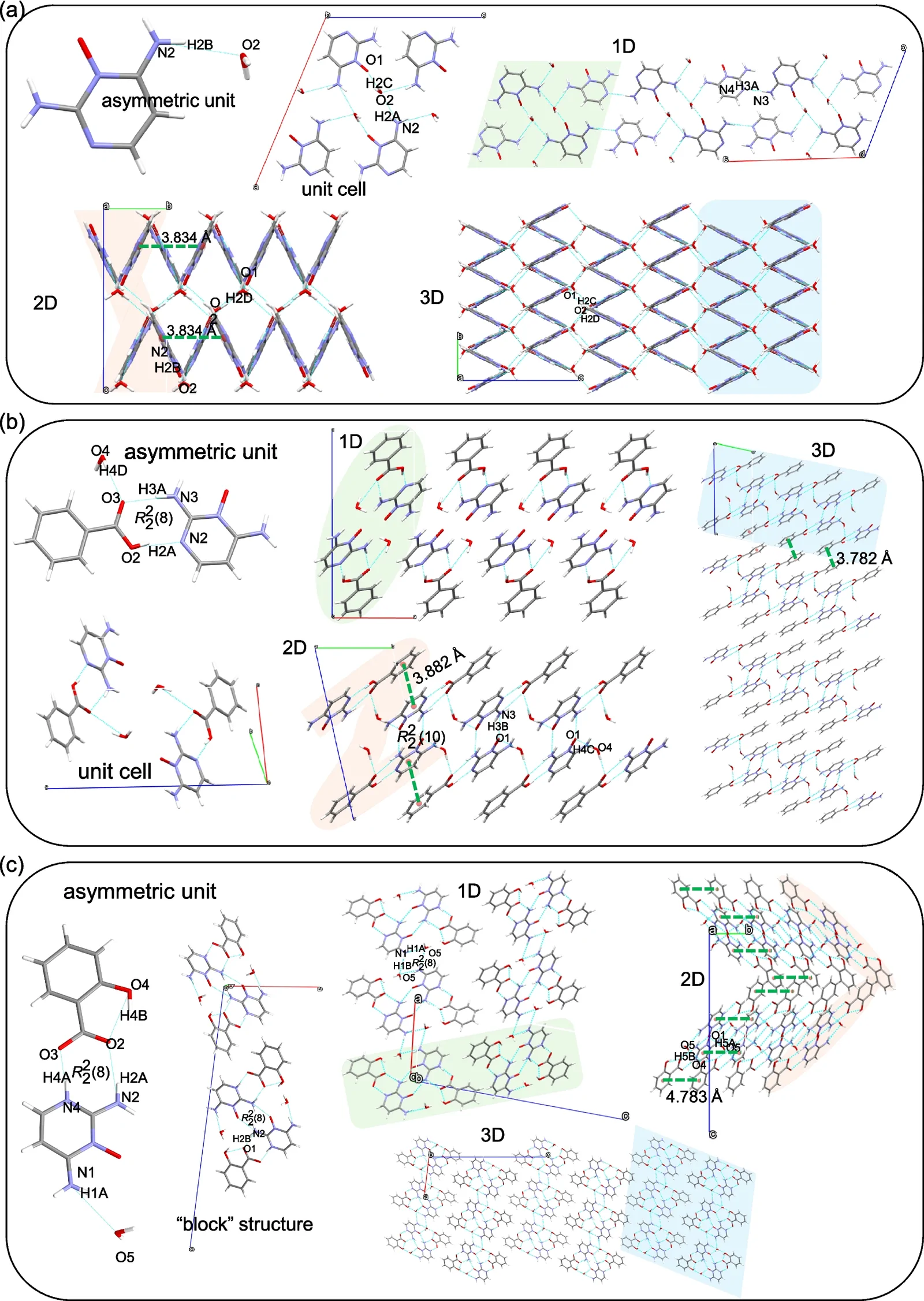
The asymmetric unit and the unit cell of (**a**) KPX·H_2_O, (**b**) KPX-BA·H_2_O and (**c**) KPX-SA·H_2_O. The 1D chain structure formed by the unit cells in green background extending along *a*-axis. The 2D layered structure is formed by 1D chains extending along *b*-axis. The part in orange background color represents the 1D chain. The 3D layered structure is formed by 2D layers extending along the *c*-axis. The part in the blue background represents the 2D layer

The key crystallographic data and refinement parameters for the single crystals of KPX·H_2_O, KPX-BA·H_2_O and KPX-SA·H_2_O are presented in Table 1. Detailed analysis of hydrogen bonding networks and molecular packing arrangements (Fig. 3) reveals distinct structural motifs, with complete hydrogen bond geometry parameters provided in Table S1. While KPX and minoxidil exhibit molecular similarity, their supramolecular behavior differs markedly. In contrast to reported minoxidil-carboxylic acid salts/cocrystals that predominately form acid-aminopyrimidine-N-oxide heterosynthons [35–39], the present KPX MCC systems exclusively manifest acid-aminopyrimidine heterosynthons (Figure S2). This distinction highlights the profound impact of subtle molecular variations on the patterns of supramolecular assembly.

**Table 1:** Crystallographic data and structure refinement parameters of KPX·H_2_O, KPX-BA·H_2_O, and KPX-SA·H_2_O

Complex	KPX·H_2_O	KPX-BA·H_2_O	KPX-SA·H_2_O
CCDC no	2430439	2430575	2430576
Empirical formula	C_4_H_8_N_4_O_2_	C_11_H_14_N_4_O_4_	C_11_H_14_N_4_O_5_
Formula weight	144.14	266.26	282.26
Temperature/K	150.15	150.15	150.15
Crystal system	monoclinic	triclinic	monoclinic
Space group	*P*2_1_/*c*	*P* $\overline{1 }$	*P*2_1_/*n*
*a*/Å	14.547(2)	6.7401(10)	9.726
*b*/Å	3.8342(5)	6.8775(10)	4.783
*c*/Å	12.1856(16)	14.927(2)	27.143
*α*/°	90	76.692(5)	90
*β*/°	112.734(4)	85.762(5)	97.08
*γ*/°	90	70.800(4)	90
Volume/Å^3^	626.87(15)	635.89(16)	1253
*Z*	4	2	4
*ρ*_calc_*g*/cm^3^	1.527	1.391	1.496
*μ*/mm^‑1^	0.124	0.108	0.12
*F* (000)	304	280	592
Radiation	MoKα (*λ* = 0.71073)	MoKα (*λ* = 0.71073)	MoKα (*λ* = 0.71073)
2*Θ* range for data collection/°	6.074 to 50.028	5.61 to 49.956	4.304 to 50.2
Index ranges	−17 ≤ *h* ≤ 17, −4 ≤ *k* ≤ 3, −14 ≤ *l* ≤ 14	−7 ≤ *h* ≤ 7, −8 ≤ *k* ≤ 8, −15 ≤ *l* ≤ 17	11 ≤ *h* ≤ 11, 5 ≤ *k* ≤ 5, 32 ≤ *l* ≤ 32
Reflections collected	7311	7889	2230
Independent reflections	1107 [*R*_int_ = 0.0526, *R*_sigma_ = 0.0376]	2177 [*R*_int_ = 0.0505, *R*_sigma_ = 0.0509]	2230 [*R*_int_ = 0, *R*_sigma_ = 0.0382]
Data/restraints/parameters	1107/0/94	2177/0/176	2230/0/185
Goodness-of-fit on *F*^2^	1.107	1.059	1.101
Final *R* indexes [I ≥ 2σ (*I*)]	*R*_1_ = 0.0522, *wR*_2_ = 0.1122	*R*_1_ = 0.0640, *wR*_2_ = 0.1453	*R*_1_ = 0.0558, *wR*_2_ = 0.1439
Final *R* indexes [all data]	*R*_1_ = 0.0862, *wR*_2_ = 0.1300	*R*_1_ = 0.1044, *wR*_2_ = 0.1673	*R*_1_ = 0.0837, *wR*_2_ = 0.1611
Largest diff. peak/hole/e Å^−3^	0.15/−0.21	0.19/−0.23	0.21/−0.34

**KPX·H**_**2**_**O** (Fig. 3a) crystallizes in the monoclinic system with space group *P*2_1_/*c* (*Z* = 4). The asymmetric unit comprises one KPX molecule and one water molecule connected via an N2-H2B···O2 hydrogen bond. Four asymmetric units assemble through N2-H2A···O2 and O2-H2C···O1 hydrogen bonds to form the unit cell. These unit cells propagate along the *a*-axis via N3-H3A···N4 hydrogen bonds, generating a 1D chain structure. Parallel chains organize along the *b*-axis through N2-H2B···O2 and O2-H2D···O1 hydrogen bonds, accompanied by π-π stacking interactions (Cg···Cg = 3.834 Å, parallel displacement), resulting in a 2D layered architecture. The 3D crystal structure completes through O2-H2C···O1 and O2-H2D···O1 hydrogen bonds between adjacent layers along the *c*-axis.

**KPX-BA·H**_**2**_**O cocrystal** (Fig. 3b) crystallizes in the triclinic system with space group *P*$\overline{1}$ (*Z* = 2), with each asymmetric unit containing neutral KPX and BA molecules, along with one water molecule. In the asymmetric unit, KPX and BA form an ${\mathrm{R}}_{2}^{2}$(8) motif through N3–H3A···O3 and O2–H2A···N2 hydrogen bonds, while BA connects to water via a hydrogen bond O4–H4D···O3. Centrosymmetric operations of asymmetric unit generate the full unit cell. Unit cells pack along the *a*-axis through van der Waals forces, forming 1D chains. Adjacent chains assemble along the *b-*axis through O4–H4C···O1 and N4–H4B···O4 hydrogen bonds, ${\mathrm{R}}_{2}^{2}$(10) motifs formed by N3–H3B···O1 hydrogen bonds, and π···π stacking (Cg–Cg distance 3.882 Å, dihedral angle 3.94°), creating 2D layers. Ultimately, the 2D layered structures with the same orientation are arranged through the π···π stacking (Cg–Cg distance 3.782 Å, parallel), thereby completing the 3D crystal structure.

**KPX-SA·H**_**2**_**O salt** (Fig. 3c) crystallizes in the monoclinic system with space group *P*2_1_/*n* (*Z* = 4) with an asymmetric unit containing protonated KPX⁺, deprotonated SA⁻, and one water molecule. The proton transfers from the carboxylic acid group of SA to the N atom of the pyrimidine group of KPX. The KPX⁺ and SA⁻ form an ${\mathrm{R}}_{2}^{2}$(8) motif through N4–H4A···O3 and N2–H2A···O2 hydrogen bonds. The KPX⁺ connects to water via N1–H1A···O5 hydrogen bond. Two asymmetric units connect via an ${\mathrm{R}}_{2}^{2}$(8) motif formed by hydrogen bonds N2–H2B···O1 to create a “hexamer”. This hexamer further assembles into a “block” structure through van der Waals forces. Neighboring block structures interconnect through ${\mathrm{R}}_{2}^{2}$(8) motifs formed by N1–H1A···O5 and N1–H1B···O5 hydrogen bonds, generating 1D chains along the *a*-axis. These chains pack along the *b*-axis through π···π stacking (Cg–Cg distance 4.783 Å, parallel) and O5–H5B···O4 and O5–H5A···O1 hydrogen bonds, generating 2D layers. The 2D layers, aligned in the same orientation, then arrange along *c*-axis through van der Waals forces, completing the 3D crystal structure.

The lattice water, HS, and BFDH analyses are displayed in Figure S3, Figures S4-7, and Figure S8, respectively.

### Solubility determination of KPX MCCs

**Fig. 4:**
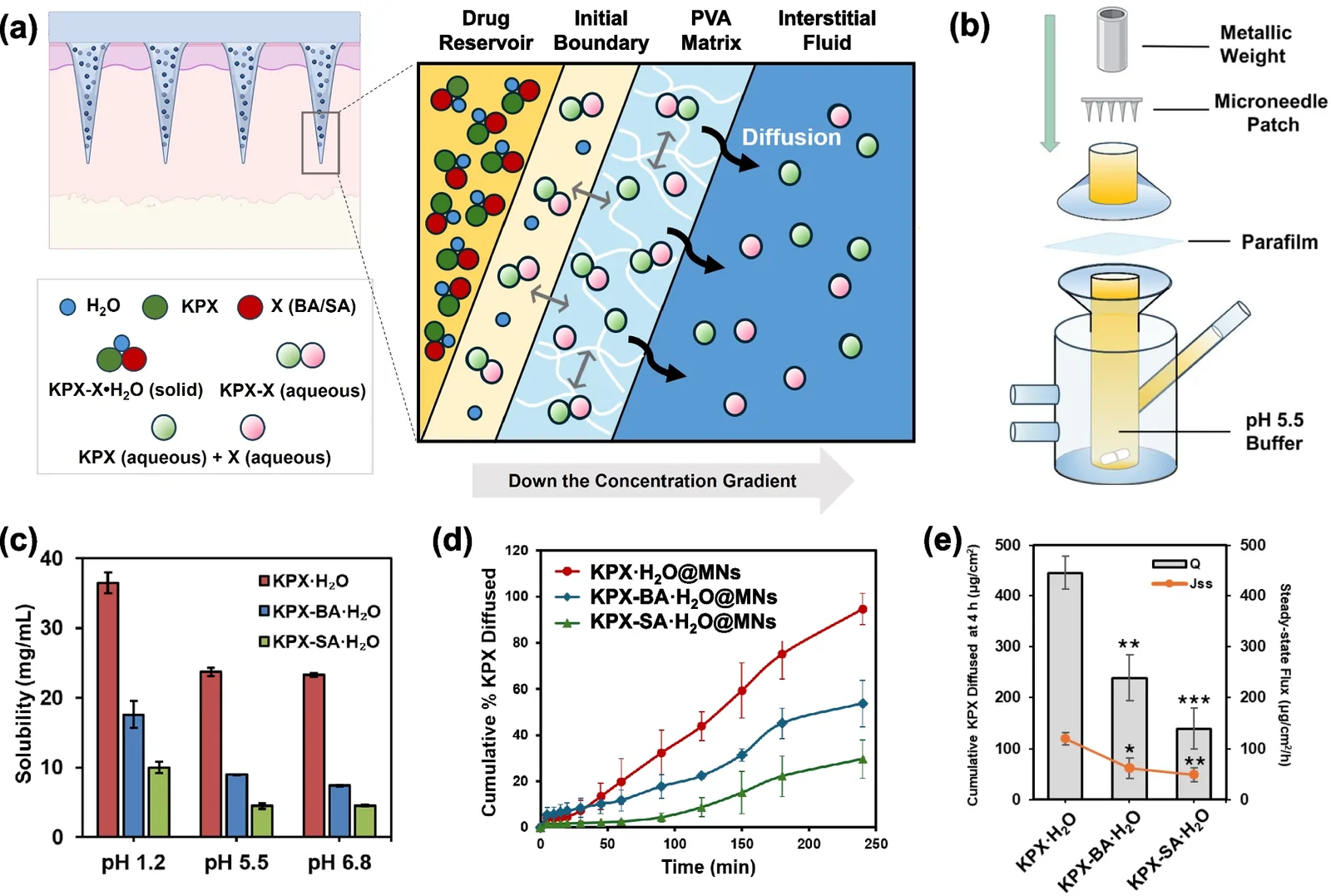
(**a**) The proposed release mechanism of KPX MCCs from MNs, (**b**) Schematic representation of the Franz diffusion cell setup for the in vitro drug release study, (**c**) Equilibrium solubilities of KPX·H_2_O, KPX-BA·H_2_O, and KPX-SA·H_2_O powders at different physiological pHs at 25 °C (data presented as mean ± SD, *n* = 3), (**d**) In vitro membrane diffusion of KPX from different MN formulations over a 4-h period (*n* = 3), and (**e**) Cumulative amounts of KPX diffused at 4 h and steady-state flux across the membrane of different MN formulations, *: 0.01 ≤ *p* < 0.05; **: 0.001 ≤ *p* < 0.01; ***: *p* < 0.001 when compared to KPX·H_2_O, *n* = 3

Unintended solution-mediated phase transformation (SMPT) of MCCs into a more stable form negates their utility as a solubility modification tool, particularly in kinetically stable systems where weak interactions between the drug and the cocrystal/salt formers fail to sustain the integrity of the crystal lattice [40–42]. In the present study, excess powders of KPX and its MCCs were suspended in pH 1.2, pH 5.5, and pH 6.8 buffer solutions to form slurry under simulated physiological conditions. Assessment of the residual solid phase after the solubility study reveals that KPX-BA·H_2_O and KPX-SA·H_2_O were thermodynamically stable over a wide pH range, with no substantial SMPT observed in the PXRD patterns (Figure S9). This implies that the MCC phases have solubilities lower than those of the drug or the cocrystal/salt former [43, 44]. Typically, the risk of phase transformation increases when the solubility of MCC surpasses that of their individual constituents [43, 44]. The HPLC analysis confirmed that the aqueous solubilities of both KPX MCCs were consistently lower than that of KPX·H_2_O across the entire range of tested pHs at 25 °C (Fig. 4c). At skin pH, i.e., 5.5, the drug solubilities of KPX-BA·H_2_O and KPX-SA·H_2_O were reduced by 2.63- and 5.25-fold compared with KPX·H_2_O, which positively correlated with the solubilities of individual constituents (BA: 3.79 mg/mL; SA: 1.89 mg/mL [45, 46]). Despite being highly soluble at gastric pH, the weakly basic KPX with a p*K*_a_ of 5.04 exhibited a pH-dependent solubility, with markedly decreased solubilities at higher pH due to diminished ionization of the amine group. As the pH shifted from 1.2 to 5.5, KPX·H_2_O showed a 34.92 ± 6.14% reduction in solubility (pH 1.2: 36.50 ± 1.52 mg/mL; pH 6.8: 23.73 ± 1.94 mg/mL), whereas KPX-BA·H_2_O (48.78 ± 1.72%) and KPX-SA·H_2_O (54.92 ± 1.92%) had a greater extent of solubility decline. The solubility–pH dependence complicates oral delivery, potentially causing erratic intestinal absorption and precipitation after gastric emptying, thereby impairing its clinical efficacy. Consequently, topical administration emerges as a more effective approach for targeted and controlled delivery of KPX to the hair follicles.

API solubility in MCCs depends on complex factors, including coformer solubility [47], crystal packing [48], hydrate formation [49, 50], stoichiometry [50, 51], molecular polarity [52], medium pH [53, 54] and common-ion effects [50, 55]. KPX-based hydrates exhibited solubilities inversely related to their melting points (KPX·H_2_O > KPX-BA·H_2_O > KPX-SA·H_2_O). The reduced solubilities are attributed to several factors. First, both BA and SA are less soluble in water than KPX. Their p*K*a values (BA: 4.20; SA: 2.98 [56, 57]) support this, as the greater acidity of SA corresponds to its lower solubility in the mildly acidic medium. Second, crystal packing, particularly the high proportion of O···H/H···O interactions contributes to the solubility differences. Although all three hydrates share similar primary intermolecular interactions (Figure S7), a higher contribution of O···H/H···O interactions tends to lower the solubility [48, 50, 58]. Finally, melting point is an insufficient indicator of solubility. Dissolution depends on two independent determinants: breaking the crystal lattice and solvating the released molecules [47, 48, 59]. In organic solvents, the solvation strength often scales with lattice strength, leading to a negative correlation between melting point and solubility. In aqueous systems, the situation reverses because solvation constraints driven by hydrophobic effects dominate over lattice energy. Therefore, melting point provides little insight into crystal aqueous solubility, where solvent–solute interactions dominate [47, 59].

### Development of KPX MCC-loaded MN patches for AGA treatment

#### Characterization of the MN patches

**Fig. 5:**
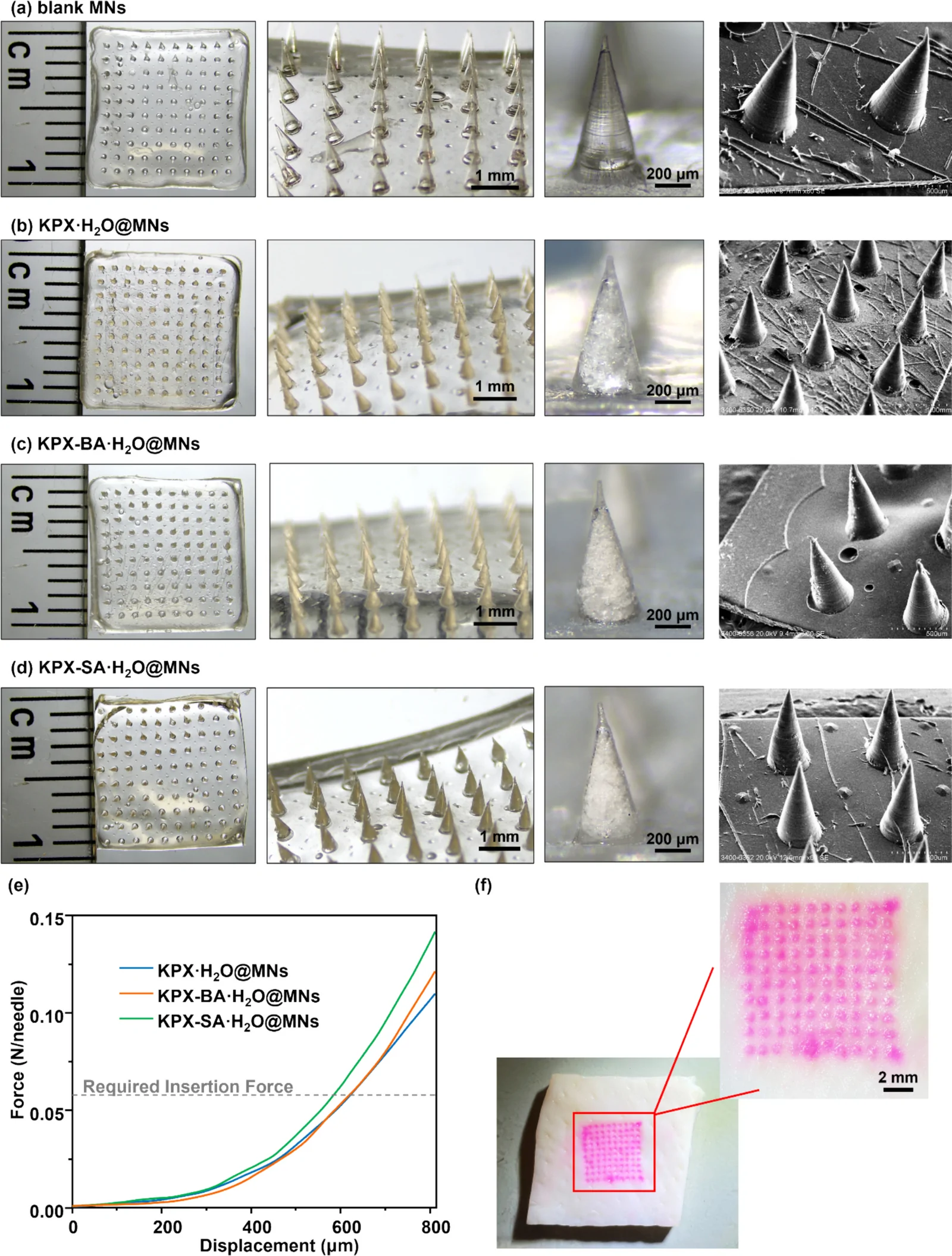
Optical microscopy and SEM images of MN patches of (**a**) blank MNs, (**b**) KPX·H_2_O@MNs, (**c**) KPX-BA·H_2_O@MNs, and (**d**) KPX-SA·H_2_O@MNs. (**e**) Compression force versus displacement curve of fabricated MNs. (**f**) Porcine skin after penetration of MN array with rhodamine B-loaded PVA shell

The biodegradable polymer solution generates hollow cone structures in the mold holes through water evaporation, which facilitates powder loading [13]. Optical microscopy analysis (Fig. 5a-d) confirmed that each fabricated MN patch comprised a uniform 10 × 10 array of microneedles arranged over a 10 mm × 10 mm area, with a center-to-center spacing of 1,000 μm. The microneedles exhibited a conical shape, with a height of 900 μm and a base diameter of 200 μm. All microneedles had sharp, well-defined tips and smooth surface topography. The MN arrays, loaded with KPX·H_2_O, KPX-BA·H_2_O and KPX-SA·H_2_O powders, were demolded carefully without structural defects such as tip breakage and absence. The powders effectively filled most of the hollow shell volume, ensuring optimal payload capacity.

To assess whether the MN patches possess the necessary mechanical strength for skin penetration under compression, the microneedles' resistance to compressive forces was evaluated. All tested patches exhibited comparable force–displacement profiles (Fig. 5e), with the drug-loaded MNs demonstrating consistent and uninterrupted force–displacement curves until reaching the predetermined displacement threshold. Previous studies suggested that a failure force of approximately 0.058 N per needle is required for successful skin penetration [60, 61]. The measured strength of the fabricated MNs could withstand the force higher than 0.1 N needle^−1^, indicating their suitability for transdermal drug delivery without structural failure upon skin insertion. To facilitate visualization during insertion, rhodamine B was incorporated into the PVA shell of the MNs. In vitro insertion tests confirmed successful penetration, demonstrating that the microneedles achieved a 100% penetration rate (Fig. 5f).

As evidenced by the PXRD data in Figure S10, the blank MNs exhibited an amorphous halo of polymer. After drug loading, KPX-BA·H_2_O@MNs and KPX-SA·H_2_O@MNs displayed weak but discernible characteristic crystalline peaks corresponding to their respective solid forms. These peaks remained detectable after one month of storage under varying conditions, confirming the physical stability of the MCC powders encapsulated within the MNs. However, KPX·H_2_O@MNs showed the peaks of anhydrous KPX, especially under 40 °C/75% RH condition, indicating that dehydration occurred. These findings further substantiate that the incorporation of MCCs in their solid-state form within the drug carrier represents a promising strategy to ensure long-term stability.

#### Drug content evaluation

The drug content in the 10 × 10 MN arrays of KPX·H_2_O, KPX-BA·H_2_O, and KPX-SA·H_2_O were determined as 1023.78 ± 16.11 μg, 471.13 ± 46.96 μg, and 483.90 ± 35.59 μg by HPLC analysis. The drug loading capacity of KPX·H_2_O@MN was ~ 2 times higher than those of the KPX-BA·H_2_O@MNs and KPX-SA·H_2_O@MNs. This disparity can be attributed to the 1:1 stoichiometric ratio between the drug and former. While clinical trials indicate high daily topical doses (90 mg) are necessary to treat alopecia [62], this is largely due to surface dripping and poor stratum corneum penetration. To accommodate the equivalent clinical dosage in the MN arrays, theoretical calculations revealed that the arrays loaded with KPX-BA·H_2_O and KPX-SA·H_2_O should exhibit needle densities of 2710 needles/cm^2^ and 2250 needles/cm^2^ with approximately 192 µm and 211 µm in pitch, assuming an average scalp surface area (SSA) of 705 cm^2^ with 1% of SSA in adults with alopecia [63–65]. However, these numbers are likely to represent overestimations for real-world applications, as the microneedle could enhance localized drug delivery by creating microchannels to bypass the stratum corneum. This strategy enables precise and direct targeting of the subdermal matrix with minimized drug loss compared to conventional topical solutions.

#### In vitro drug release profiles and drug deposition

As effective treatment requires release in a bioavailable state, this study utilized a specialized Franz diffusion cell setup [30] to characterize sub-milligram KPX release in a low-volume medium simulating skin condition (Fig. 4b). While Parafilm M^®^ does not replicate the aqueous, porous nature of dermal-epidermal tissue, it is employed here primarily as a model barrier to compare relative release rates.

The cumulative percentage of drug diffused through the membrane was plotted against time (Fig. 4d). KPX-BA·H_2_O@MNs (*p* = 0.0260) and KPX-SA·H_2_O@MNs (*p* = 0.0054) exhibited significantly slower drug diffusion at pH 5.5 (skin pH) compared with KPX·H_2_O@MNs over a period of 4 h. At the end point of the study, KPX·H_2_O@MN delivered a 445.74 ± 32.01 μg/cm^2^ of KPX, corresponding to nearly complete release of the total drug content in the patch. In contrast, KPX-BA·H_2_O@MNs and KPX-SA·H_2_O@MNs delivered amounts of KPX that were 1.86-fold and 3.20-fold lower, respectively, than those delivered by KPX·H_2_O@MNs at 4 h. The steady state flux (*J*_SS_) of each MN was estimated by the best-fit slopes of the apparent linear portions (R^2^ > 0.97). Several initial data points were excluded from the fitting process [33, 66]. The values of *J*_SS_ were 120.84 μg/cm^2^/h, 62.12 μg/cm^2^/h, and 49.37 μg/cm^2^/h for KPX·H_2_O@MNs, KPX-BA·H_2_O@MNs, and KPX-SA·H_2_O@MNs, respectively (Fig. 4e and** S11**). The lag time to reach steady-state concentration (*T*_lag_) was determined by extrapolating the linear segment of each steady-state permeation curve to the intercept on the time axis. Drug penetration through the membrane possessed a short *T*_lag_ of 12 min when delivered in the form of KPX·H_2_O@MNs, which extended to approximately 1 h when formulated as KPX-SA·H_2_O@MNs. However, statistical analysis of *T*_lag_ indicated no significant difference between KPX-BA·H_2_O@MNs and KPX·H_2_O@MNs (*p* = 0.79).

The diffusion rate of the MN arrays correlated positively with solubility of the KPX MCC at pH 5.5, following the trend: KPX·H_2_O > KPX-BA·H_2_O > KPX-SA·H_2_O (Fig. 4c). Upon skin insertion, the hydrophilic PVA microneedle shell absorbed water, triggering gradual degradation and bulk erosion of the polymeric matrix over time. Water infiltration into the matrix created a thin hydrated interface, i.e., aqueous initial boundary layer, between the inner lining of the microneedle cavities and the dry KPX MCC solid powders embedded within (Fig. 4a). The drug powders in the MNs first underwent dissolution in the initial boundary layer generating a highly supersaturated region, subsequently diffused into the polymeric matrix and released into the receptor compartment. This process is largely dependent on the solubility and dissolution rate of the encapsulated dry powders. In the initial boundary layer, KPX-BA·H_2_O and KPX-SA·H_2_O with higher packing efficiencies but lower solubilities than KPX·H_2_O slowly transformed into solubilized crystals. In other words, MCC formation indirectly retarded KPX diffusion by reducing the concentration of soluble crystals within the initial boundary layer, thereby limiting the availability of solubilized drug for diffusion into the polymeric matrix and ultimately bulk aqueous medium, attaining a sustained release effect. As the PVA continues to erode over time, water infiltrates more freely into the central cavity, allowing the remaining core of powder to hydrate. Ultimately, the entire structure (shell and powder core) hydrates and dissolves as a single, integrated unit, with the erosion rate of the PVA matrix controlling the overall release profile. To explore the KPX diffusion mechanism of different MN arrays, the data were fitted into four common kinetic models: zero-order, first-order, Higuchi, and Korsmeyer–Peppas models (Figure S12). The zero-order kinetic model best described the drug diffusion from the KPX·H_2_O MN across the 4-h study (R^2^ > 0.99), implying constant release. Notably, after the formation of cocrystal hydrate and salt hydrate, the drug release shifted to the first-order kinetic model. The change can be ascribed to a multifaceted interplay of factors, such as drug solubility, drug-polymer interaction capability, and degradation of the matrix, etc. [67] Specifically, the highly water-soluble KPX·H_2_O molecules have a strong propensity to readily form hydrogen bonds with the hydroxyl groups of the PVA matrix during diffusion, resulting in uniform dispersion within the polymeric structure and a release profile predominantly governed by the steady dissolution of the matrix. In contrast, the more hydrophobic KPX MCC molecules exhibited reduced binding affinities with PVA. Due to a very high density of vacant polymer binding sites relative to the local free drug present, the release process followed first-order kinetics with drug retention depending on the amount of free drug present [68].

For the 12-h drug deposition study, no significant difference was observed in the percentage of drug deposited in porcine skin among the groups: KPX·H_2_O@MNs (24.18 ± 2.82%), KPX-BA·H_2_O@MNs (25.40 ± 5.25%), and KPX-SA·H_2_O@MNs (26.29 ± 5.43%). This result can be attributed to the MN-mediated bypass of the stratum corneum barrier, which minimizes the impact of solubility and permeability differences on intradermal drug delivery. Extended therapeutic delivery is crucial for managing chronic conditions like alopecia. While current microneedle strategies rely on nanoparticles or biodegradable polymers [69, 70], cocrystal engineering has emerged as effective for sustaining release by modifying solubility through enhanced lattice energies and hydrophobic interactions [71–73]. However, cocrystal-loaded microneedle patches have received only limited attention. Our findings demonstrate that integrating cocrystals with microneedle technology optimizes KPX delivery, reducing dosing frequency and skin irritation by preventing concentration spikes [74]. Future investigation on the dose selection, in vivo pharmacokinetic, and safety profiles is warranted to facilitate clinical translation.

### Study limitations

While this study demonstrates the potential of MN arrays for enhanced intradermal delivery of solid-state MCCs, several limitations should be acknowledged. First, Parafilm M^®^ was employed as a skin alternative for in vitro drug release, despite its structural and physicochemical differences from biological tissue. As a hydrophobic, non-porous thermoplastic, Parafilm M^®^ lacks the viscoelasticity, hydration, and collagen fiber architecture characteristic of the human stratum corneum and dermis [31]. Consequently, it cannot fully replicate moisture-induced polymer dissolution that occurs in vivo. Second, the in vitro release profile of KPX·H_2_O@MN exhibited near-complete KPX release within approximately 4 h, which is not aligned with the chronic nature of AGA, a condition that typically requires sustained drug exposure [1, 2]. In contrast, KPX-BA·H_2_O@MNs and KPX-SA·H_2_O@MNs, which exhibited slower release kinetics, appear more suitable for maintaining therapeutic levels over extended periods. Clinical translation would therefore require either increased dosing frequency or modifications of the MN matrix to prolong drug release. Lastly, a translational gap remains between the in vitro extraction concentrations observed in this study and the localized tissue concentrations expected in vivo. The current experimental setup was conducted under static diffusion conditions, whereas in vivo drug disposition is strongly influenced by cutaneous blood perfusion, lymphatic drainage, and metabolic clearance. Future studies utilizing in vivo models are required to characterize the precise steady-state concentrations of KPX under conditions of dynamic vascular clearance.

## Conclusion

This study presents the rational design and synthesis of the first MCCs of the anti-alopecia agent KPX. The formation of KPX-BA·H_2_O (cocrystal hydrate) and KPX-SA·H_2_O (salt hydrate) has been confirmed through comprehensive solid-state characterizations, including PXRD, DSC, TGA, FTIR, and SCXRD. Both structures feature acid–aminopyrimidine heterosynthons and share a 1:1:1 stoichiometry between KPX, the coformer, and water. The KPX MCCs were thermodynamically stable when suspended in aqueous solutions over a wide physiological pH range, of which the solubilities exhibited a distinct structure–property relationship dependent on the crystal packing arrangement. The MCC powders were further encapsulated into 10 × 10 array of MNs that possessed desirable morphological characteristics, mechanical strength, and drug loading capacity, thereby substantiating their suitability for potential transdermal hair regrowth therapy. The integrity of KPX MCC powders was well-preserved within MNs after one-month storage under accelerated conditions. Compared with KPX·H_2_O, KPX-BA·H_2_O and KPX-SA·H_2_O achieved significantly slower drug diffusion from MNs in a Franz diffusion cell model, with a 1.86-fold (*p* = 0.0260) and 3.20-fold (*p* = 0.0054) reduction in the amounts of KPX delivered at 4 h, respectively. The diffusion of the KPX MCCs was positively correlated with their solubilities at pH 5.5, following the first-order kinetic model. Given the lack of reports on KPX MCCs, these results can provide guidance on MCC development for APIs yet to be explored. Our work has demonstrated the feasibility of fabricating MCC-based microneedle formulations with sustained drug diffusion and satisfactory physical stability.

In translational contexts, BA and SA are recognized as pharmaceutically accepted coformers with extensive prior human use, thereby facilitating safety assessment and regulatory review processes. Furthermore, the minimally invasive microneedle patch permits self-application and enhances patient adherence. These features offer a translational opportunity for formulating AGA as a drug-device combination product. This strategy could serve as an appealing alternative to address existing treatment challenges across a range of dermatological conditions.

## Supplementary Information

Below is the link to the electronic supplementary material.

## Data Availability

The datasets generated during and/or analysed during the current study are available from the corresponding author on reasonable request.

## Abbreviations

ACN: Acetonitrile
AGA: Androgenetic Alopecia
APIs: Active Pharmaceutical Ingredients
BA: Benzoic Acid
BFDH: Bravais–Friedel–Donnay–Harker
DHT: Dihydrotestosterone
DSC: Differential Scanning Calorimetry
EtOH: Ethanol
FDA: Food and Drug Administration
FIM: Full Interactions Map
FTIR: Fourier-Transform Infrared
GRAS: Generally Recognized as Safe
HPLC: High Performance Liquid Chromatography
HS: Hirshfeld Surface
KPX: Kopexil
MCC: Multicomponent Crystal
MeOH: Methanol
MEP: Molecular Electrostatic Potential
MN: Microneedle
PDMS: Polydimethylsiloxane
PVA: Polyvinyl Alcohol
PXRD: Powder X-Ray Diffraction
SA: Salicylic Acid
SCXRD: Single Crystal X-Ray Diffraction
SEM: Scanning Electron Microscope
SMPT: Solution-mediated Phase Transformation
SSA: Scalp Surface Area
TGA: Thermogravimetric Analysis
VMD: Visual Molecular Dynamics
