# Supplementary material for: Dissolving microneedles incorporating kopexil multicomponent crystals for improved transdermal delivery
Source: Drug Deliv Transl Res. 2026 Jan 28;16(9):3351–67. doi: 10.1007/s13346-025-02042-0 (PMC13538242; doi:10.1007/s13346-025-02042-0)
Supplement: Supplementary file 1 [file 13346_2025_2042_MOESM1_ESM.docx]

**Supplementary Information**

**Dissolving Microneedles Incorporating Kopexil Multicomponent Crystals for Improved Transdermal Delivery**

Yuehua Deng ^a, b^, Si Nga Wong ^a, b^, Wing Chi Nico Chan ^a^, Xinyue Zhang ^a^, Shing Fung Chow ^a, b*^

^a^ Department of Pharmacology and Pharmacy, Li Ka Shing Faculty of Medicine, The University of Hong Kong, Pokfulam, Hong Kong SAR, China

^b^ Advanced Biomedical Instrumentation Centre, Hong Kong Science Park, Shatin, New Territories, Hong Kong SAR, China

*^*^* *Corresponding author*

**Table S1.** Hydrogen-bond geometry (Å) of KPX·H_2_O, KPX-BA·H_2_O, and KPX-SA·H_2_O.

|  | ***d*(D–H)/Å** | ***d*(H···A)/Å** | ***d*(D···A)/Å** | **(D–H···A)/°** |
| --- | --- | --- | --- | --- |
| **KPX·H_2_O** |  |  |  |  |
| N2–H2A···O2 | 0.880 | 2.132 | 2.939 | 152.07 |
| N2–H2B···O2 | 0.880 | 2.068 | 2.921 | 162.95 |
| O2–H2C···O1 | 0.870 | 1.878 | 2.745 | 174.25 |
| O2–H2D···O1 | 0.870 | 2.024 | 2.823 | 152.06 |
| N3–H3A···N4 | 0.880 | 2.159 | 2.997 | 158.90 |
| **KPX-BA·H_2_O** |  |  |  |  |
| N3–H3A···O3 | 0.880 | 1.953 | 2.816 | 166.30 |
| O2–H2A···N2 | 0.840 | 1.772 | 2.596 | 166.32 |
| O4–H4D···O3 | 0.869 | 2.143 | 2.962 | 156.64 |
| N3–H3B···O1 | 0.880 | 2.010 | 2.760 | 142.38 |
| O4–H4C···O1 | 0.871 | 1.828 | 2.697 | 176.20 |
| N4–H4B···O4 | 0.880 | 1.980 | 2.858 | 174.99 |
| **KPX-SA·H_2_O** |  |  |  |  |
| N2–H2A···O2 | 0.881 | 2.058 | 2.916 | 164.50 |
| N2–H2B···O1 | 0.880 | 2.180 | 2.895 | 138.08 |
| N4–H4A···O3 | 0.880 | 1.688 | 2.560 | 171.01 |
| O5–H5A···O1 | 0.870 | 1.979 | 2.795 | 155.80 |
| N1–H1A···O5 | 0.881 | 2.201 | 2.997 | 150.12 |
| N1–H1B···O5 | 0.880 | 1.990 | 2.853 | 166.49 |
| O5–H5B···O4 | 0.870 | 2.139 | 2.951 | 154.85 |
| O4–H4B···O2 | 0.840 | 1.835 | 2.575 | 146.00 |

**Figure S1.** The overlaid TGA thermogram of KPX·H_2_O and its MCCs. Weight loss of dehydration for KPX·H_2_O, KPX-BA·H_2_O, and KPX-SA·H_2_O are 12.8%, 7.5%, and 7.0%, respectively.

**Figure S2.** Heterosynthons in (a) KPX-BA·H_2_O and (b) KPX-SA·H_2_O.

**Lattice water analysis**

Water molecules present in crystalline organic compounds can be categorized as either lattice water or channel water [1]. In the three crystals examined in this study, all water molecules are identified as lattice water, participating in well-defined intermolecular interactions with the organic components. These hydrate structures feature water motifs characterized by a "DDAA" hydrogen-bonding configuration [2], where each water molecule acts as both two hydrogen bond donors (DD) and two hydrogen bond acceptors (AA). **Figure S3** illustrates the distinct water interaction networks in each hydrate. In KPX·H_2_O, every water molecule forms four hydrogen bonds exclusively with adjacent KPX molecules. In KPX-BA·H_2_O, each water molecule interacts with three KPX molecules and one BA molecule. Similarly, in KPX-SA·H_2_O, water bridges three KPX⁺ and one SA⁻. These interactions highlight the critical role of lattice water as a structural nexus, facilitating multidimensional molecular connectivity and enhancing the stability of the crystal packing through directional hydrogen bonds.

**Figure S3.** Water interaction maps for the water molecules in (a) KPX·H_2_O, (b) KPX-BA·H_2_O, and (c) KPX-SA·H_2_O.

[1]	Ahlqvist MUA, Taylor LS. Water Diffusion in Hydrated Crystalline and Amorphous Sugars Monitored Using H/D Exchange. J Pharm Sci 2002;91:690-98.

[2]	Gillon AL, Feeder N, Davey RJ, Storey R. Hydration in Molecular CrystalsA Cambridge Structural Database Analysis. Cryst Growth Des 2003;3:663-73.

**HS analysis**

HS analysis was performed to characterize the nature of intermolecular interactions within the supramolecular lattices. The HS visualization employs a color-coded scheme where red regions indicate contacts with distances shorter than the sum of van der Waals radii (*r*_vdW_), blue regions represent contacts exceeding *r*_vdW_, and white areas denote distances equal to *r*_vdW_. The surfaces are plotted as a function of the normalized contact distance (*d*_norm_), defined through the parameters *d*_e_ (distance to the nearest external nucleus) and *d*_i_ (distance to the nearest internal nucleus).

The 3D HS along with 2D fingerprint plots for KPX·H_2_O, KPX-BA·H_2_O, and KPX-SA·H_2_O are shown in **Figures S4-S6**, with quantitative contributions of intermolecular contacts detailed in **Figure S7**. Analysis of the contact histograms reveals that the structural stability of KPX·H_2_O predominately arises from H···H (43.8%), N···H/H···N (19.0%), O···H/H···O (14.8%) and C···H/H···C (8.6%). The N···H/H···N contact is contributed by both H_2_O-KPX, and KPX-KPX interactions, while the H···O/O···H contact mainly stems from H_2_O-KPX interactions. It is noteworthy that H⋯H contacts constitute the largest contribution to the HS in three crystal structures. After the incorporation of coformer and H_2_O, the contribution of both H···H and N···H/H···N decrease. In contrast, the contribution from the strong interactions O···H/H···O increase substantially from 14.8% in KPX·H_2_O to 26.0% in KPX-BA·H_2_O and 32.3% in KPX-SA·H_2_O, becoming the secondary predominant type of interaction. This O···H/H···O contact enhancement is associated with the formation of acid-aminopyrimidine supramolecular heterosynthons between KPX and BA/SA. The KPX MCC formation alters the KPX crystal lattice arrangement, resulting in an increased availability of effective hydrogen bond sites with varying degrees of enhancement.

**Figure S4.** HS mapped according to *d*_norm_, along with two-dimensional fingerprint plots of KPX·H_2_O.

**Figure S5.** HS mapped according to *d*_norm_, along with 2D fingerprint plots of KPX-BA·H_2_O.

**Figure S6.** HS mapped according to *d*_norm_, along with two-dimensional fingerprint plots of KPX-SA·H_2_O.

**Figure S7.** Percentage contribution of molecular contact interactions to the HS in KPX·H_2_O, KPX-BA·H_2_O and KPX-SA·H_2_O.

**A** comparison between the BFDH-predicted crystal morphologies and experimental SEM images for the three crystalline forms in **Figure S8** illustrates that both KPX·H_2_O and KPX-SA·H_2_O crystals feature elongated prismatic morphologies, while KPX-BA·H_2_O adopts a rhombohedral habit. The close correspondence between the calculated and observed crystal shapes validates the morphological predictions, showing strong agreement between theoretical modeling and experimental results.

**Figure S8.** BFDH calculated crystal morphologies and SEM images of (a) KPX·H_2_O, (b) KPX-BA·H_2_O, and (c) KPX-SA·H_2_O.

**Figure S9.** No major phase transformation of the KPX MCCs was detected when suspended in different physiological pH buffers at equilibrium.

**Figure S10.** PXRD patterns of (a) KPX·H_2_O@MNs, (b) KPX-BA·H_2_O@MNs and, (c) KPX-SA·H_2_O@MNs after one month of storage under varying conditions.

**Figure S11.** Quantification of the permeation parameters: the steady-state flux is the slope of the linear part of the graph of the cumulative amount penetrated as a function of time; the lag time is the time intercept of the linear portion of the graph.

**Figure S12.** Kinetic analysis of drug release: A) zero-order, B) first-order, C) Higuchi, and D) Kosermayer–Peppas kinetic models (**□** KPX·H_2_O; **△** KPX-BA·H_2_O; **⨯** KPX-SA·H_2_O).
